# Autophagy in Disease Onset and Progression

**DOI:** 10.14336/AD.2023.0815

**Published:** 2024-08-01

**Authors:** Hao Wang, Xiushen Li, Qi Zhang, Chengtao Fu, Wenjie Jiang, Jun Xue, Shan Liu, Qingxue Meng, Lisha Ai, Xuejun Zhi, Shoulong Deng, Weizheng Liang

**Affiliations:** ^1^Shenzhen Baoan Women's and Children's Hospital, Jinan University, Shenzhen, Guangdong, China.; ^2^Department of Obstetrics and Gynecology, Shenzhen University General Hospital, Shenzhen, Guangdong, China.; ^3^School of Medicine, Huzhou University, Zhejiang, China.; ^4^Department of Artificial Intelligence and Data Science, Hebei University of Technology, Tianjin, China.; ^5^Department of General Surgery, The First Affiliated Hospital of Hebei North University, Zhangjiakou, Hebei, China.; ^6^Bioimaging Core of Shenzhen Bay Laboratory Shenzhen, China.; ^7^Technology Department, The First Affiliated Hospital of Hebei North University, Zhangjiakou, Hebei, China.; ^8^Department of Teaching and Research, Shenzhen University General Hospital, Shenzhen, Guangdong, China.; ^9^Department of Respiratory and Critical Care Medicine, The First Affiliated Hospital of Hebei North University, Zhangjiakou, Hebei, China.; ^10^National Health Commission of China (NHC) Key Laboratory of Human Disease Comparative Medicine, Institute of Laboratory Animal Sciences, Chinese Academy of Medical Sciences and Comparative Medicine Center, Peking Union Medical College, Beijing, China.; ^11^Central Laboratory, The First Affiliated Hospital of Hebei North University, Zhangjiakou, Hebei, China.

**Keywords:** autophagy, infection, disease, cancer, liver, kidney, heart, ovary, lung, brain, bowel, blood

## Abstract

Autophagy is a biological phenomenon whereby components of cells can self-degrade using autophagosomes. During this process, cells can clear dysfunctional organelles or unwanted elements. Autophagy can recycle unnecessary biomolecules into new components or sometimes, even destroy the cells themselves. This cellular process was first observed in 1962 by Keith R. Porter et al. Since then, autophagy has been studied for over 60 years, and much has been learned on the topic. Nevertheless, the process is still not fully understood. It has been proven, for example, that autophagy can be a positive force for maintaining good health by removing older or damaged cells. By contrast, autophagy is also involved in the onset and progression of various conditions caused by pathogenic infections. These diseases generally involve several important organs in the human body, including the liver, kidney, heart, and central nervous system. The regulation of the defects of autophagy defects may potentially be used to treat some diseases. This review comprehensively discusses recent research frontiers and topics of interest regarding autophagy-related diseases.

## 1. Introduction

In eukaryotes, there are two main molecular degradation pathways: the autophagy system and the ubiquitin-proteasome system. By degrading redundant, dysfunctional, and damaged cellular components, these two systems jointly maintain the balance of intracellular material balance. Although recent evidence has suggested that there is some crosstalk between the two degradation pathways, they are still essentially considered to have different biological functions [1, 2]. Compared with the ubiquitin-proteasome system, autophagy mainly degrades long-lived and macromolecular proteins in the cytoplasm, as well as degrades damaged organelles. Autophagy is a metabolic process in which cells encapsulate their proteins and organelles in a particular membrane, and then deliver them to lysosomes for degradation to produce energy and small molecules, (e.g., amino acids), which are available for cellular reuse. This is imperative for the cell to tolerate starvation, clear incorrectly folded proteins, and repair impaired aging organelles as well as for sustaining intracellular homeostasis. This process is present in a very wide range of eukaryotic cells, from single-celled yeasts to multi-cellular mammals. Autophagy is an important regulator of eukaryotic cell homeostasis.

In this review, we summarized the mechanisms behind autophagy in different disease states and clarify the effects of autophagy on disease generation and progression, which will hopefully help the field to formulate new therapeutic strategies and contribute to new insights for the development of novel disease treatment strategies.

**Figure 1.**
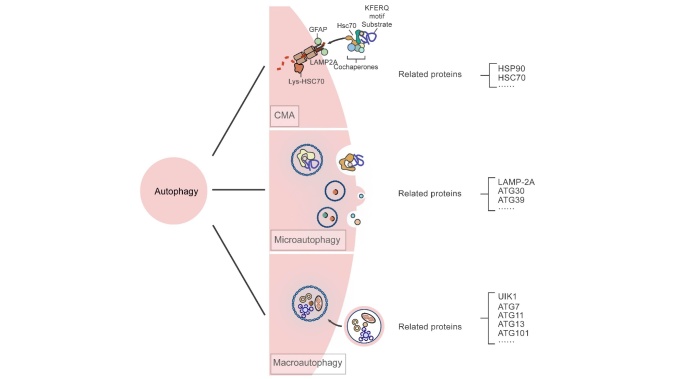
The main classification of autophage and related important proteins.

Based on whether the process makes use of the lysosomal or the vacuolar pathway of substrate binding, autophagy is conventionally classified into the following three major forms: macro-autophagy, micro-autophagy, and chaperone-mediated autophagy (CMA) [3] (Fig. 1).

### 1.1 Macroautophagy

**Figure 2.**
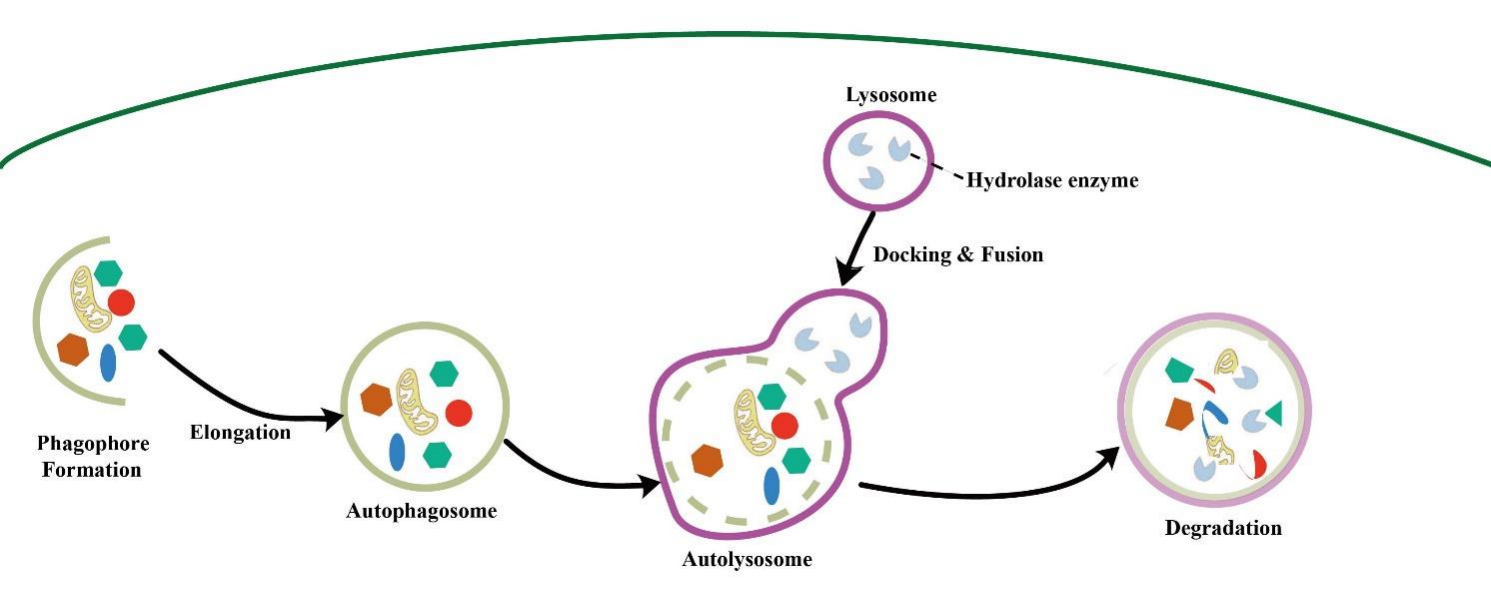
The flow of macroautophagy.

There are three general types of autophagy, and the difference between the different types lies in how the cargoes are transported into the lysosome. Macroautophagy, as one of these, maintains cellular metabolism and homeostasis by capturing and degrading intracellular proteins and organelles [4] (Fig. 2). In the macroautophagy pathway, cargoes are sequestered in double-membrane autophagosomes, and mature autophagosomes are degraded by fusion with lysosomes [5]. Macroautophagy is thought to have a "cell-cleaning" function, removing proteins and damaged organelles that may contribute to the pathogenesis and progression of disease [6]. In neurodegenerative diseases, intracellular accumulation of damaged proteins leads to neuronal dysfunction and death, while macroautophagy halts disease progression by degrading the recipient protein [7, 8]. Lymphatic endothelial cells induce tolerance in T cells by presenting peripheral tissue antigens, and macroautophagy is an important factor affecting lymphatic endothelial cell immune function under inflammatory conditions [9]. Autophagy occurs in the membrane of the endoplasmic reticulum (ER) and is activated by the ULK1 complex and class III PI3K (PI3KC3) complex I (VPS34-Beclin 1-ATG14-AMBRA1-p115) when the ULK1 complex triggers the PI3KC3 complex through phosphorylation. The PI3KC3 complex initiates PI3P production at the ER subdomain, forming a structure called the omegasome. In parallel to the PI3P provided by the PI3KC3 complex, ATG9-containing vesicles from other apoplastic membranes are also engaged in autophagy priming. Beginning in the omegasome structure, a series of reactions that produce massive amounts of ATG will take place to permit the autophagosome to elongate, seal, mature and integrate with the lysosome [10, 11].

Over 30 ATGs have now been discovered. Since 2003, autophagy genes in yeast have been named uniformly. ATGs are used to indicate autophagy-specific genes and proteins [12]. Human autophagy-related genes have homologous counterparts, but with distinct names. Cells often form crescent-shaped bilayers in the cytoplasm when exposed to stress conditions such as starvation and hypoxia. These bilayer membranes are called phagophores and are generally considered to be important markers indicating the beginning of autophagy. The bilayer membrane may come from the mitochondrial membrane, ER membrane, Golgi membrane, or cytoplasmic membrane [13-15]. Phagophore formation in mammals is usually induced by the ULK1 protein kinase complex, which can be regulated by AMPK and mTOR. ULK1 is mainly used to regulate the extension of the isolation membrane, and its activity is proportional to the size of the membrane, but its reaction must be combined with that of dephosphorylated ATG13 [16]. ATG13 can be phosphorylated in yeast and mammals under nutrient-rich conditions. By contrast, if nutrient deficiency occurs, yeast ATG13 is rapidly and entirely dephosphorylated, while in mammalian cells it is only partially dephosphorylated [17]. FIP200 has no clear homology in yeast, but it is considered to be the functional counterpart of ATG7 and ATG11 in yeast [18]. The interaction between ULK1 with ATG13 and FIP200 contributes to the correct localization of ULK1 and the enhancement of its activity and stability [19]. ATG101 is involved in maintaining the stability of ATG13 and ULK1, the autophosphorylation of ULK1, and the recruitment of PI3P with regard to binding to the isolation membrane.

### 1.2 Microautophagy

Over the past 50 years, studies on microautophagy have mainly focused on yeasts, with few focusing on mammalian cells [20]. The main mechanism behind mammalian microautophagy is that the lysosomal membrane forms arm-like or valve-like bulges that engulf part of the cytoplasm or damaged organelles. This is known as the lysosomal encapsulation mechanism. Details of the underlying mechanism behind this process have not yet been clarified in mammals [21, 22]. Microphagocytes can usually be divided into selective, non-selective, and endosomal-microphagocyte. Microphagy itself can be divided into three types according to membrane dynamics: the lysosome concave type, the lysosome protruding type, and the inner body concave microphagocytic type [21]. The lysosome membrane encapsulates the degradation products into lysosomes through depression, which is considered to be related to multiple autophagy-related proteins and the delivery of the endosome sorting composite [23, 24]. Endosomal microautophagy is also associated with multivesicular bodies, including the HSC70 and Nbr1 pathways [23, 25].

### 1.3 Chaperone-mediated autophagy

Molecular CMA is a specific lysosomal debasement procedure that involves the recognition of cytoplasmic proteins with certain motifs by molecular chaperones and binding to special receptors, such as LAMP-2A, on the lysosomal membrane. LAMP-2A is a single transmembrane lysosomal membrane protein with only 12 amino acid residues on the cytoplasmic side and a high glycosylation activity in the cavity of the lysosome. CMA can be divided into the following steps: (1) substrate recognition and lysosome targeted transportation. (2) binding to the substrate and unfolding; (3) substrate entering the lysosome and degrading. Autophagy substrates are first mediated by molecular chaperones that generally have KFERQ-like sequences, which are recognized and bind to the molecular chaperone Hsp70, which facilitates protein transmembrane transport. Hsp70 then forms complexes with other molecular chaperone proteins such as Hsp90, Hsp40, HOP, HIP, and Bag-1 in the process of molecular chaperone-mediated autophagy. Once the identified substrate binds to the molecular chaperone complex, the substrate is targeted to the lysosomal surface and binds to LAMP-2A, and after which it is transported into the lysosomal cavity for degradation [26, 27].

Both macroautophagy and microautophagy can proceed via selective or non-selective autophagy, depending on the target. In contrast to the non-selective autophagy model that has been discussed thus far in this review, there are also several common selective autophagy models such as the Cvt pathway (cytoplasmic to vacuolar targets pathway), peroxisomal autophagy, mitotic autophagy, and endoplasmic reticulum autophagy. These selective autophagic processes are similar to the non-selective autophagy process; nevertheless, selective autophagy is distinguished by its specific substrate recognition processes. The receptor protein binds to the ligand to be degraded and forms an aggregate, which then forms specific autophagosomes in vesicles and bilayers near the receptor protein or its scaffolding protein. The receptor for mitochondrial autophagy in yeast, for instance, is its outer membrane protein Atg32 [28], which can bind to other Atg core proteins to make damaged aging mitochondria enter the autophagosome. Most autophagy events belong to the non-selective autophagy class.

The occurrence of autophagy has been described in the below steps. (1) Autophagosomes form specific structures such as preautophagosomal structures (PASs; precursor structures of autophagosomes) in yeast cells and recruit other Atg proteins and membrane components such as vesicles for localization. Omega as well as separation membranes then form cup-like structures in mammalian cells. (2) The double-layered membranes of autophagosomes can expand, extend, and encapsulate various intracellular components under the modulation of multiple Atg proteins. (3) These bilayers extend and close with the assistance of Atg proteins to form autophagosomes. (4) The external membrane of the autophagosomes fuse with those of lysosomes or yeast vacuoles, forming special structures known as autophagic lysosomes, while the internal membranes and their contents decay into small molecules under the actions of esterases and proteases. (5) Autophagic lysosomes produce tubular structures with the assistance of membrane-related proteins such as Clathrin, Kif5b, and Dynamin, to form new lysosomes through autophagosome regeneration [29, 30]. Macroautophagy is the most important type of autophagic process. Its main molecular mechanism is discussed in the following sections.

### 1.4 The Atg1 complex

Atg1 which was defined as the first autophagy gene identified in yeast cells is also a serine/threonine kinase [31]. The ATG1 complex comprises Atg13 and Atg1, which are its core components, and the dephosphorylation of Atg13 can induce autophagy. Several upstream kinases involved in the autophagy process can regulate the phosphorylation of the Atg1/ULK complex, such as the target of the rapamycin (Tor) kinase complex. AMP-activated protein kinase (AMPK), and protein kinase A (PKA), that can phosphorylate Atg1 and Atg13. In a nutrient-rich environment, the Tor complex phosphorylates Atg13 extensively. Under starvation or other external stress conditions, however, Tor kinase is inactivated and Atg13 is dephosphorylated. As a result, Atg13 can bind to Atg1, activating its kinase activity. Atg1 then forms dimers and self-phosphorylates [32]. According to a recent report, Atg13 can bind to Atg1 regardless of its phosphorylation state. However, when autophagy occurs, the dephosphorylation of Atg13 greatly enhances its binding to Atg1, and it combines with the Atg17-Atg31-Atg29 scaffold complex of the PAS structure to form PAS, after which it recruits other Atg proteins [33]. The homologous protein of Atg1 kinase in mammalian cells is ULK1/2. The ULK1/2 complex comprises ULK1/2 and ATG13 (homologous to yeast Atg13), FIP200 (homologous to yeast Atg17), and ATG101. The Atg1/ULK complex exerts a critical effect during the initiation stage of autophagy [34].

### 1.4 The PI3K class complex

In addition to the Atg1 complex, the PI3K complex also exerts an important signal transduction function in autophagy [35]. The PI3K complex includes Atg14, Vps34, Vps15 and Atg6 (homologous to mammalian Becline1). Beclin1 binds to Vps34 and UVRAG (homologous to yeast Vps38) to form a complex that induces autophagy [36]. This complex can phosphorylate PIP2 to generate PIP3 and guide downstream Atg proteins to localize onto the PAS structure, the autophagy precursor in mammalian cells, which can in turn bind to bilayer membrane components and promote autophagy [37]. These downstream proteins include Atg18 and Atg21. Atg18 can form the Atg2-Atg18 complex and plays a role in the Atg9 cycle.

### 1.5 The WIPI-Atg2 Complex

PI3P can recruit a variety of effectors from the WD-repeat (WDR) protein network, as well as dual FYVE-containing protein 1 (DFCP 1) [38]. WIPI (a WD-repeat protein that interacts with phosphoinositides) is a member of the WDR family that interacts with PI3P. At present, four WIPI proteins have been reported: WIPI1, WIPI2, WlPI3, and WIPI4 [39]. The homolog of WIPI1 and WIP2 in yeast is Atg18. WIPI family proteins can specifically recognize a segment of Atg2 protein (the WIR-Motif) to form the WIPI-Atg2 complex that then translocates to the junctions of phagocytic vesicles and the ER, to promote the extension and closure of autophagosomes [40].

### 1.6 Atg9

Atg9 is highly conserved evolutionarily and is the only transmembrane protein involved in the autophagy process. Atg9 is located on monolayer vesicles in specific regions of the cytoplasm and can be seen as a repository of Atg9 in the cytoplasm. When autophagy occurs, Atg9 vesicles are transported back and forth between PASs and the cytoplasmic repository[41]. Atg9 vesicle localization onto PAS or autophagosomes requires assistance from Atg17, Atg11, Atg23, and Atg27. Leaving the vesicles requires activation of the Atg1 (ULK1/2) and Atg2-Atg18 (WIFI) complexes [42]. The specific mechanism behind this, however, has not yet been fully clarified. It is currently believed that Atg9 vesicles provide the initial membranes or protein compositions for the formation of autophagosome bilayers.

**Figure 3.**
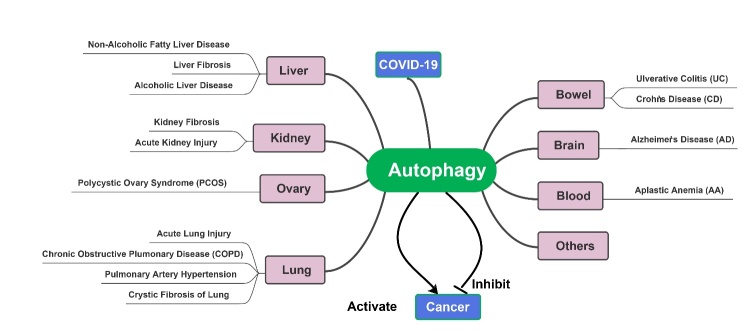
Autophagy is involved with the occurrence and development of several diseases.

### 1.7 Ubiquitin-like Conjugation Systems in Autophagy

The extension of the autophagosome membrane involves two ubiquitin-like proteins (Atg12 and Atg8/LC3) and two related conjugation systems [43, 44]. One of the pathways covalently connects Atg12 to Atg5 under the action of E1-like ligase Atg7 and E2-like ligase Atg10, to form PAS along with Atg16L1. In the second ubiquitination pathway, Atg8/LC3 is first cut by protease Atg4, after which phosphatidylethanolamine is covalently linked to the cut Atg8/LC3 under the action of the E1-like ligase Atg7 and the E2-like ligase Atg3. This process leads to the transformation of LC3 from LC3-I to LC3-II. LC3-II can bind to the newly formed autophagosomal membrane and facilitate its fusion with the lysosome. LC3-II is therefore commonly used as a marker of intracellular autophagy [45].

Autophagy is partially responsible for the overall maintenance of homeostasis in the endocellular environment, as well as the disposal of some ubiquitinated protein polymers that are not degraded by the proteasome. Disorders in the autophagy system can result in severe problems for cells. It has been found in mice that autophagy disorders contribute to cell degeneration, age-related changes, tumor formation, and harmful infections [3]. Autophagy may have a crucial role to play in some human diseases, which has been proven in several studies [46-49](Fig. 3). Autophagy has long been thought to be closely associated with human disease, but because autophagy in humans cannot be precisely measured, there is still little evidence that autophagy activity increases or decreases under specific conditions. Over the past decade, however, human genetic studies have increasingly shown that autophagy is associated with a multitude of conditions, especially neurodegenerative diseases, cancer, inflammatory diseases, and autoimmune diseases. Autophagy-related gene mutations are known to cause Mendelian disease, and autophagic polymorphism is associated with susceptibility to certain diseases. In this review, we will discuss the relationships between autophagy and diseases that occur in several major human organs. Understanding the functions of autophagy in these diseases can help us better study and treat them. Hopefully, this information is helpful in terms of providing new ideas for the development of novel drugs to treat some of these autophagy-related diseases.

## 2. The Central Nervous System

### 2.1. Alzheimer’s Disease

The most common form of aging-associated dementia is Alzheimer's disease(AD), which accounts for 50-75% of dementia cases [50]. The onset of AD is insidious, and mainly occurs in the elderly or pre-elderly stage. Its symptoms typically manifest as memory disorders, cognitive decline, personality and behavior changes, and general declines in the ability to perform daily living tasks [51]. Exotic age spots (SPs) consisting of β-amyloid (Aβ) accumulation and intracellular neurofibrillary tangles (NFTs) consisting of abnormally phosphorylated tau protein accumulation comprise the prototypical pathological changes in AD [52]. It has been suggested that autophagy abnormalities are closely connected to the initiation and advancement of some neurodegenerative diseases including AD.

Autophagy is indispensable for maintaining neuronal homeostasis [53]. It is of particular importance to maintain normal autophagic function in neurons, as they are permanent cells[54]and cannot be diluted by other cells to divide the toxic materials that they produce. Defective autophagy is responsible for the aberrant build-up of proteins such as Aβ and abnormal phosphotau-like proteins, which comprise two of the main pathological changes in AD senile plaques and nerve fiber entanglement. Hara et al. [55] and Komatsu et al. [56] noticed that abnormal accrual of ubiquitinated proteins, massive shrinkage of neurons, and behavioral deficits in mice within weeks after Atg5 or Atg7 were knocked out. This experiment proved that autophagy abnormalities are tied to the onset and development of AD.

One of the major ingredients of age spots is Aβ, generated by the β-secretase and γ-secretase lysis of precursor protein (APP). APP is a type I transmembrane protein that is distributed extensively in various membrane architectures, such as the axons, and dendrites of neurons [57, 58]. Located in the cell membrane, APP can be biodegraded by both amyloid and non-amyloid proteins. The non-amyloid protein degradation pathway refers to the cleavage of APP into a soluble fragment called sAPPα, which can be released into the extracellular space, and α-CTF (the C terminal fragment), which continues to bind to the plasma membrane under the action of α-secretase. The amyloid degradation pathway refers to the formation of hydrolysis product p3 under the action of γ-secretase. Some APPs also enter the cell lumen through endocytosis to form endosomes. The β- and γ-secretases are abundant in the endosome. Compared with α-secretase, β-secretase can cleave APP closer to the N-terminal into sAPPβ and β-CTF, and γ-secretase can further degrade β-CTF into Aβ [59]. When the endocytosis pathway or the endocytosis of APP is selectively blocked, the generation of Aβ is significantly decreased.

Yu et al. found that autophagic vesicles (AVs) contain a large number of APPs, β-CTF, and BACE in the liver cells of mice that overexpressed APP, suggesting that AVs may represent potential sites for Aβ formation [60]. Subsequent studies have confirmed that AD patients have AVs in their brains that contain high levels of APP, AV, and γ-secretase complexes, and that the activation of autophagy occurs in the earlier phase of AD, resulting in the amplification of AVs and the generation of large amounts of Aβ [61]. After oxidative stress was induced in SH-SY5Y cells that overexpressed APP *in vitro*, autophagy levels increased markedly, and there was a pronounced rise in the number of AVs that contained APP, Aβmonomers, and oligomers. 3-MA was able to significantly lower the Aβ levels in the cells. These studies have demonstrated that autophagy is a critical way to generate Aβ. AVs not only decompose APP that has been encapsulated by AVs into Aβ, but also transport βCTF from endosomes to autophagosomes, which are then diluted by γ-secretase to yield significant quantities of Aβ [62].

Yuan et al. found that proteins related to autophagy such as Atg5, Beclin1, and Ulk1 are strongly linked to Aβ and APP-CTF degradation. After Beclin-1 in N2a-APP cells was knocked down using siRNA, the levels of Aβ and APP-CTF in cells increased significantly [63]. Jaeger et al. confirmed that the substrates related to autophagy contained APP and their degradation products, while accumulated APP and CTF typically correlated with impaired clearance of autophagosomes [64]. By contrast, SMER28 (one of the autophagic agonists) and rapamycin have significantly enhanced the clearance of intracellular CTF and Aβ [63].

The impairment of Aβ elimination is a major contributor to the pathology of AD. AVs, containing Aβ, are typically required to be transported to and degraded by lysosomes, which carry essential cathepsins like cathepsins B and D capable of breaking down Aβ [65, 66]. This is because lysosomes are primarily scattered around the pericaryon, meaning that AVs formed in neurite outgrowths need long-distance reverse transport to fuse with lysosomes [67]. Large numbers of autophagosomes, loaded with APP and related secretases, were found to have accumulated in swollen axons in the cerebral cortexes and hippocampi of AD patients and AD manikin mice [68], indicating hindered elimination of AVs in AD brains. Conserved AVs are unable to be digested by lysosomes, which contributes to the intracellular accumulation of Aβ and the acceleration of pathological AD events. It is worth noting that although both pathways responsible for Aβ degradation (the proteasome pathway and the lysosome pathway) are impaired in AD, compared with rodents, human seems to rely more on the lysosome pathway to eliminate Aβ produced in the brain[69].

Autophagy is capable of regulating both the synthesis as well as the removal of Aβ and bears a role in its secretion as well. Nilsson et al. found that knocking down Atg7 in APP transgenic mice dramatically lowered the secretion of Aβ and the number of extracellular senile plaques [70]. Infection with lentivirus, bearing Atg-7, later enabled Atg7 to return to normal levels and reactivate autophagy, restoring Aβ secretion to its prior levels. In wild-type mice whose junior neurons were exposed to low-grade rapamycin administration, there was a significant increase in the formation of intracellular autophagosomes, in conjunction with increased secretion of Aβ. By contrast, the inhibition of autophagy by spautin-1 significantly reduces the secretion of Aβ.

AVs have been found deposited in malnourished neurite outgrowths in the brains of AD patients, increasing the chance of fusion with the plasma membrane which in turn leads to more Aβ escaping cells as extracellular amyloid plaques. Extracellular Aβ, however, is not the main cause of AD. One study demonstrated that raising intracellular Aβ content led to perception disorder in AD model mice in the absence of extracellular amyloid plaques [71]. Tomiyama et al. found that not only was an increase in intracellular Aβ oligomers able to induce changes in synchronization, but it could also lead to the abnormal phosphorylation of the tau protein without the presence of senile plaques. This suggested that extracellular amyloid plaques are not necessary for the pathogenesis and development of AD and that intracellular Aβ is the causative agent [72]. The intracellular accumulation of Aβ not only interferes with autophagosomal transport and its fusion with lysosomes, but also destabilizes the lysosomal membrane, affects the substrate decomposition of autophagy, and further aggravates the pathological changes of AD.

One protein that is mainly distributed throughout the axons of neurons in the middle cerebral system is the tau protein, which is a microtubule-binding protein [73]. Normally, the tau protein promotes microtubule assembly and binds to it to maintain its stability, facilitating the axonal transport of “molecular cargo” [74]. There are at least 30 phosphorylation sites on the tau protein. Normally, most of them are dephosphorylated [75]. However, in the brains of AD patients, the amount of phosphorylated tau protein can increase by 4-8 fold [76]. These abnormally phosphorylated tau proteins not only lose their normal function, but also form neurofibrillary tangles that are not easily degraded by proteolytic enzymes, accumulate around the nucleus, and accelerate cell death [73]. The main phosphoenzyme of the tau protein is phosphoenzyme 2A (PP2A) [77]. Dysfunction of PP2A directly leads to over-phosphorylation of the tau protein, encourages its self-agglomeration and the formation of deposits, and eventually exacerbates pathological changes in AD [78]. Autophagy inhibitors can block tau protein degradation and accelerate the formation of NFTs [79-81]. The phospholipase D1 (PLD1) blocks autophagosomal ripening and accelerates the accumulation of tau proteins in the brain, which act as negative modulators of Vps34 [82]. Berger et al. found that rapamycin was able to reduce the toxicity of the tau protein in an AD *Drosophila* model by increasing its hydrolysis [83]. Significantly increased amounts of LC3-II and autophagosomes were seen upon treatment with the proteasomal inhibitor MG-132, while cellular levels of the tau protein decreased significantly [84, 85]. This was likely due to a radical activation and upregulation of the autophagic lysosomal pathway in response to the blocking of the proteasome pathway, in turn accelerating tau protein hydrolysis. By contrast, degradation of the tau protein as a result of arresting the autophagic pathway of neurons through the ubiquitin-proteasome pathway (UPS) was found to be minimal [86]. This demonstrated that, compared with the proteasome pathway, the primary method used to degrade the tau protein in neurons is autophagy. It is also worth noting that tau protein fragments of different molecular weights are degraded by different autophagy routes. For example, tauRDΔK280 is a truncated tau protein, which is mainly degraded via the CMA pathway [81]. A typical accumulation of the tau protein can in turn impede autophagic events. The hyperphosphorylation of the tau protein abolishes its ability to assemble the microtubule, which in turn retards the reverse transport of axons and autophagosome-lysosome fusion. Ultimately, autophagosomes accumulate in neurons [87, 88], which hinders the removal of abnormal proteins and further aggravates the pathological processes behind the development of AD.

## 3. The Respiratory System

### 3.1. The Lung

Autophagy is known to be associated with some pulmonary conditions and serves a vital role in lung infections, acute lung lesions, interstitial lung fibrosis, chronic obstructive pulmonary disease, and an array of other pertinent disorders.

#### 3.1.1 Acute Lung Injury

Acute Lung Injury (ALI) is an acute hypoxic change in non-cardiovascular origin caused by a number of reasons. Its pathophysiological characteristics include lung volume reduction, pulmonary compliance reduction, and an imbalance of ventilation flow-ratio [89, 90]. Mechanical ventilation is one of the most common causes of ALI. Patients with severe respiratory failure need mechanical ventilation, but long-term exposure to high concentrations of oxygen can lead to lung injury. It has been found that LC3B regulates hyperoxia-induced apoptosis of lung epithelial cells. Under high oxygen conditions, more LC3-I is converted to LC3-II. Studies have found that LC3B is closely related to Fas, an apoptosis-inducing gene. The interaction between p62, LC3B, and caspase-8 substrate Atg342 may be the key to understanding how LC3B regulates the Fas signaling pathway [91]. The autophagy signaling pathway involved in the development of ALI induced by hyperbaric oxygen provides potential therapeutic targets.

Alveolar epithelial cells (AECs) include types 1 and 2 (AEC1 and AEC2). As the progenitor cells of the alveolar epithelium, AEC2 cells can synthesize and secrete alveolar surface-active substances that can enhance lung tissue function and effectively sustain the stability of the alveolar structure, and its self-renewal following lung tissue apoptosis [92]. Liu et al. reported increases in the levels of autophagy-related proteins LC3 and Beclin-1, as well as hyperinflammatory factors IL-6 and TNF-α, in the AEC2 cells of mice treated with LPS, which suggested that SIRT1 may participate in autophagy by affecting Atg7 in AEC2 cells, thereby protecting mice from infection [93]. Fan et al. have also shown that inflammatory signaling pathways are regulated by autophagy in case of AEC injury or lung ischemia-reperfusion injury, and that rapamycin can act as an autophagy promoter to alleviate the lung inflammation caused by lung ischemia-reperfusion injury [94]. However, there have also been studies showing that the deletion of the *Akap1* gene in mice can lead to excessive autophagy in AEC2 cells, so that the lung is exposed to hyperoxia conditions, contributing to an abundance of inflammatory factors and aggravating lung injury in mice. It can thus be seen that when autophagy is properly activated in AEC cells, it can be used as an adaptive response to sustain the relative homeostasis of the endocellular system and protect the lung tissue. However, excessive autophagy, by contrast, may aggravate lung tissue damage.

AMPK is an important metabolic regulator that can restore energy balance under metabolic stress and regulate inflammation. Its metabolic regulation is closely related to that of mTOR. Activation of AMPK can inhibit mTOR activity and stimulate autophagy. When the AMPK/ mTOR signaling pathway is activated, ACE2 can alleviate LPS-induced ALI inflammation and the severity of lung injury caused by excessive autophagy in rats. Autophagy inhibitor 3-methyladenine can also reduce the severity of ALI in mice, suggesting that autophagy plays a crucial role in LPS-induced ALI.

Based on the results of animal studies, Fan et al. found that the decrease in AMPK dephosphorylation induced by LPS may lead to the enhancement of mTOR activation and the inhibition of autophagy, thus promoting the development of LPS-induced inflammatory injury [95]. Adenosine kinase activators can restore LPS-induced autophagy inhibition, IL-6 elevation, and lung histological abnormalities, as well as improve the survival rates of mice following LPS treatment. This suggests that AMPK dephosphorylation-mediated activation of the mTOR pathway may be involved in LPS-induced lung inflammation in mice by inhibiting excessive autophagy.

The PI3K/AKT/mTOR signaling pathway is the primary signaling approach responsible for regulating autophagy in mammalian cells under specific conditions (such as infection, aging, and oxidative stress). Chang et al. found that HeLa cells were able to resist coxsackievirus B3 infection by increasing the expression of autophagy marker LC3 and the formation of autophagysomes [96]. PI3K/AKT/mTOR signaling pathway may be involved in the autophagy processes of HeLa cells following viral infections. Inhibiting this pathway could therefore affect the autophagy reaction caused by the viral infection, resulting in changes in the inflammatory reactions of the lung tissue after contraction. This suggests that PI3K/AKT/mTOR is an important intracellular signaling pathway that is regulated by autophagy. Cong et al. reported that fine particles can activate the PI3K/AKT/mTOR signaling pathway by up-regulating IL-17A, to inhibit the autophagy of bronchial epithelial cells, and promote lung inflammation and fibrosis [97]. Studies have shown that hydrogen sulfide can inhibit autophagy through the PI3K/AKT/mTOR pathway, to effectively improve the pathological changes induced in lung tissues by lipopolysaccharides, in turn reducing the expression of inflammatory factors, and preventing ALI in mice [98]. Cystic fibrosis transmembrane conductance regulators can also inhibit autophagy through the PI3K/AKT/mTOR pathway, reducing LPS-induced ALI in mice [99]. Therefore, autophagy can be inhibited by activating the PI3K/ AKT/mTOR signaling channel, thereby reducing lung injury.

Studies have shown that Bcl-2 can regulate mitochondrial mitosis in LPS-induced ALI by inhibiting the PINK1/Parkin signaling pathway and thereby reducing the degree of autophagy in lung tissue, as well as reducing the overall inflammatory response [100]. Araya et al. found that human lung epithelial-like cells A549 activated PINK1/Parkin signaling pathway-mediated mitophagy during paraquat-induced apoptosis [101]. Paraquat, during the process, induced PINK1/Parkin-mediated mitosis, and the inhibition of Parkin gene expression via transient transfection with Parkin small interfering RNA (siRNA) was able to reduce Paraquat-induced autophagy and exacerbate apoptosis in A549 cells. By contrast, the overexpression of Parkin was able to attenuate paraquat-induced cell injury through the promotion of mitosis. These results suggest that PINK1/Parkin-mediated mitosis has a protective function in paraquat-induced epithelial-like A549 cell injury in the lungs.

#### 3.1.2 Chronic Obstructive Pulmonary Disease

Chronic obstructive pulmonary disease (COPD) is among one of the most widespread pulmonary diseases in seniors and is mainly characterized by incompletely reversible airway ventilation limitation and small airway remodeling [102]. The expression of autophagy-related proteins (e.g., LC3, Beclin1, Atg4, Atg7, Atg5-Atg12) has been documented to be significantly elevated in the lung tissues of patients with COPD [103]. Cigarette smoking is the main cause of COPD, and LC3-I to LC3-II conversion is one of the most important indicators that can be used to measure the degree of autophagy. Chen et al. found that autophagosomes increased along with LC3-II expression in the lung tissues of rats who had been raised long-term in the presence of cigarette smoke [104]. Chen et al. also found that autophagy is substantially higher in the lung tissues of patients with COPD caused by congenital defects in α1-AT [104]. Jeffrey A added cigarette extract to a culture of alveolar epithelial cells and alveolar macrophages *in vtiro*, to establish a smoking model. After the cigarette extract intervention, there were markedly greater numbers of autophagosomes found in the alveolar epithelial cells compared with the control group, and LC3-II was visibly upregulated as well [105]. Studies have shown that cigarette extracts can induce alveolar epithelial cell death by activating the expression of the death-inducing signal complex (DISC) and its downstream apoptotic protease targets. After silencing the autophagy genes *Beclin-1* and *LC3B*, the apoptosis of cells pretreated with cigarette extract was inhibited, indicating that the autophagy-related protein Beclin-1 and LC3B can effectively regulate the apoptosis of alveolar epithelial cells caused by smoking [106]. Fas is an important component of DISC. Research has found that LC3B forms a complex with Fas and is regulated by caveolin-1. LC3B quickly dissociates from Fas in the presence of cigarette extracts, in a similar manner to the activation of the apoptosis pathway. Further studies have found that caveolin-1 mutation inhibits the expression of LC3B [107]. Chen et al. observed that both cells and rats with caveolin-1 gene deletions had stronger autophagy fluxes [107]. The expression of Egr-1, a transcription factor that binds to the LC3 promoter region, is significantly enhanced under stress and inflammation. Chen et al. found that the expression of Egr-1 in the alveolar epithelial cells of rats exposed to cigarette smoke was increased [104]. The expression of LC3B in alveolar epithelial cells after Egr-1 gene silencing was inhibited by cigarette extract. In animal experiments, the pro-apoptotic and autophagic levels of Egr-deficient rats exposed to smoke were significantly decreased, and the alveolar expansion caused by smoking was significantly inhibited. These results indicate that autophagy is tightly connected with COPD, but whether the mechanism of autophagy causing alveolar enlargement is due to its promoting of alveolar epithelial cell apoptosis or through other signaling pathways is unclear.

#### 3.1.3 Pulmonary Artery Hypertension

Pulmonary hypertension refers to a pulmonary artery mean pressure of>25 mmHg at rest or > 30 mmHg during exercise [108, 109]. The expression of LC3 is elevated in the lungs of patients with pulmonary hypertension, and the conversion rate of LC3-Ⅰ to LC3-II is also significantly increased, especially in the endothelial cell layer and the outer and middle layers of the resistance vessels. LC3-silenced mice are more likely to develop pulmonary hypertension under chronic hypoxia in animal experiments [110]. This suggests that LC3B may be involved in and inhibit the formation of pulmonary hypertension. However, Tanaka et al. performed experiments on fetal sheep and found that removing the autophagy gene *Beclin-1* impaired angiogenesis in continuous pulmonary artery endothelial cells. These experiments show that *Beclin*-1-mediated autophagy may promote the progression of pulmonary hypertension [111]. Therefore, the involvement of autophagy is crucial for the treatment of pulmonary hypertension.

Pulmonary hypertension is firmly tied to mitochondrial malfunction. In patients with pulmonary hypertension, the intracellular glucose oxidation pathway is inhibited; the glycolysis pathway is activated, and mitochondrial function is weakened, which in turn can weaken cell oxidative stress and inhibit cellular apoptosis in the short term [112]. However, if mitochondrial function continues to be inhibited, vascular remodeling is promoted [113]. One study found that the amount of mitochondrial fragmentation and mitochondrial fission proteins present in the pulmonary artery smooth muscle cells of patients with pulmonary hypertension was significantly elevated. This process is called mitophagy and still needs to be further studied in terms of its relationship to pulmonary hypertension.

#### 3.1.4. Cystic Fibrosis

Cystic fibrosis, arising from mutations to the regulator of cystic fibrillation, the transmembrane transduction (CFTR), is a deadly hereditary disease [114, 115]. Its most characteristic manifestation is dyspnea caused by long-term repeated pulmonary infections [116]. The most common CFTR mutation is a gene deletion encoding phenylethylamine in the 508 coding region[117]. Bence et al. found that in patients with cystic fibrosis patients who harbor this mutation, autophagy pathway defects, intracellular polyubiquitinated protein accumulation, and autophagosome clearance all decreased [118]. CFTR deficiency leads to the accumulation of reactive oxygen species (ROSs) in cells and the up-regulation of glutamine transferase2 (TG2) expression. TG2 further causes cross-linking and inactivation of Beclin-1, which inhibits the portion of the autophagy pathway mediated by the protein, in turn causing sequestosome-1 (p62) to accumulate in cells [119]. Abdulrahman et al. found that the infection rate of Burkholderia was significantly decreased in mice after using an autophagy agonist, and that the inflammatory response in lung tissues was also reduced [120]. Enhanced autophagic activity may therefore represent a novel target for the management of pulmonary cystic fibrosis.

### 3.2. The Liver

Liver diseases often develop into chronic diseases with high mortality rates that seriously affect the safety and quality of life of patients. It has been well-documented that autophagy is closely correlated with various liver conditions. Moderate autophagy bears the stamp of protection, while excessive or dysfunctional autophagy boosts cell death and leads to the germination and advancement of liver disorders. One potential remedy for liver disease, therefore, may involve the modulation of autophagy.

#### 3.2.1. Non-Alcoholic Fatty Liver Disease

Nonalcoholic fatty liver disease (NAFLD) has rapidly grown into a major cause of liver disease worldwide, accompanying such global epidemics as obesity and diabetes [121-123]. It is also a leading cause of surgical liver transplantation operations. NAFLD can be divided into two major types: non-alcoholic fatty liver (NAFL) and non-alcoholic steatohepatitis (NASH). NASH is typically more serious than NAFL. Over 90% of obese people and 60% of diabetic people develop NAFLD [121, 122].

Studies have reported that the expression of both ATG and TFEB significantly decreases in NASH patients, as well as in mice fed a high-fat diet. Conversely, rubicon (an autophagy inhibitor) and p62 start to increase [124-126]. A decreased level of autophagy has also been observed in the hepatic endothelia of patients with NASH. In mice cells, the interruption of the autophagy pathway, such as through the depletion of Atg7 in hepatocytes, and the depletion of Atg5 in endothelial cells, causes the exacerbation of many of the features of NAFLD [127, 128]. These features include lipid droplet accumulation, hepatocyte injury enhancement, and increased ER stress. By contrast, the external delivery of Atg7 to mice can improve their statuses with regard to the status of liver injury and steatosis, as well as decrease ER stress.

The recommended lifestyle therapies for NAFLD include dietary modification and physical activity. However, some recent studies have found that FDA-approved drugs for NAFLD such as digoxin, ikarugamycin, and alexidinedihydrochloride can promote TFEB nuclear translocation and enhance autophagic flux. The drugs that are capable of improving lipid metabolism, insulin resistance, and fat deposition in mice caused by a high-fat diet are thought to be TFEB agonists [129]. DeBosch et al. found that metformin targeting AMPK has promising anti-steatogenic effects [130]. Caffeine has also been found to have similar effects on stetatosis [131]. The regulation of autophagy is therefore useful for the treatment of NAFLD, since lipophagy has an anti-steatogenic function in hepatocytes. Mitophagy can confer hepato-protective effects on patients with NASH, as macrophages have anti-inflammatory properties.

#### 3.2.2. Liver Fibrosis

Chronic liver parenchymal cell injury is associated with liver fibrosis. The primary cause behind the initiation of the fibrotic process in the liver is the activation of hepatic stellate cells (HSCs) [132]. A critical marker that represents the activation of HSCs is the disappearance of lipid droplets. The digestion of lipid droplets by autophagy is considered to serve as the energy source that drives the activation of HSCs. Studies have found that the inhibition of autophagy can downregulate the fibrogenic status of HSCs [133, 134].

The regulatory effects of autophagy on liver fibrosis are extensive and complex, capable of both promoting and inhibiting it. Hepatic fibrosis is triggered by the activation of HSCs, and the elimination of lipid droplets is an important indicator of the activation of HSCs. Autophagy channels lipid droplets through degradation and the presence of autophagosomes, which in turn efficiently clears excess lipid droplets. Lipid droplet digestion during autophagy is thought to be a source of energy for the activation of HSCs, which in turn promotes liver fibrosis. Autophagy can also inhibit fibrosis, as it has been reported that the inhibition of apoptosis triggered by the autophagy-mediated miR-30a-5p/ATG5 axis alleviates liver fibrosis [135]. The autophagy inhibitor chloroquine also inhibits the activation of HSCs and thereby exerts anti-fibrosis effects [136]. Liver fibrosis is a reversible disease. Although current research has a certain understanding of its occurrence and the mechanism behind its development, the function of autophagy in relation to hepatic fibrosis is poorly understood.

#### 3.2.3. Alcoholic Liver Disease

Alcoholic Liver Disease (ALD) is a chronic liver injury triggered by long-term excessive drinking, whose pathogenesis is extremely complex. ALD can develop into alcoholic hepatitis, liver fibrosis, cirrhosis, and even liver cancer. ALD has become a serious public safety and health concern [137]. Significant evidence has shown that the mechanism behind ALD pathogenesis is related to alcohol-induced aberrant autophagy. Improving autophagy levels can therefore partially alleviate alcoholic liver injury.

Lu et al. [138] found that autophagy alleviates liver injury by reducing the oxidative stress induced by CYP2E1 in ethanol-fed mice. The inhibition of autophagy by drugs or siRNAs targeting Atg7 can significantly increase hepatocyte apoptosis and liver injury [139]. Although the mechanism whereby autophagy prevents the development of alcoholic liver injury is not very clear, some studies have suggested that it is mainly related to the clearance of impaired mitochondria, along with excess lipid droplets, through autophagy. This means that mitophagy and lipophagy play important protective roles in ALD. Cytophagy is a process of selective autophagy that can degrade injured or excessive mitochondria. The PINK1-Parkin signaling pathway is closely related to mitophagy. In normal cells, the PINK1 protein is synthesized in the cytoplasm,translocated to the inner mitochondrial membrane, and cleaved by the preserved protein-associated rhodopsin (PARL) protease in the intima, which is then quickly degraded by the ubiquitin-proteasome [140]. However, PINK1 is neither cleavage cleaved nor degraded when mitochondria are damaged, contributing to its accumulation on the outer mitochondrial membrane. Subsequently, PINK1 in the outer membrane is then activated by autophosphorylation, and the cytoplasmic Parkin protein is recruited to mitochondria as a result. Next, activated PINK1 phosphorylates and activates Parkin E3’s ubiquitin ligase activity. Autophagosomes are then recruited to mitochondria, and damaged mitochondria are degraded through another cascade of reactions [141]. Williams et al. found that Parkin was able to prevent alcoholic liver injury in mice exposed to ethanol. The group also found that, compared with wild-type mice, both mitochondrial lesions and oxidative stimulation were dramatically increased in the livers of Parkin knockout mice [142]. They also found that after treatment with ethanol, mitophagy, β-oxidation, and cytochrome c oxidase activities in Parkin knockout mice were lower than they were in wild-type mice. This evidence proved that Parkin could alleviate liver injury to a certain extent by mediating autophagy to remove damaged mitochondria.

Lipophagy is a type of selective autophagy that delivers the contents of lipid droplets to lysosomes for degradation with the assistance of autophagosomes, effectively removing excess lipid droplets. Unlike adipose tissue, the liver is usually considered a fat-burning organ, not a fat-storage organ. Therefore, lipid autophagy is of great significance in preventing lipid droplet accumulation in hepatocytes and maintaining lipid homeostasis. Schulze et al. found that ethanol drinking significantly reduced the activity of Rab7, a small guanosine triphosphatase in hepatocytes, thereby damaging the transport and fusion between autophagy machine and lipid droplets, and resulting in hepatic steatosis [143]. Lin et al. found in the ALD model that after the hepatocytes were activated by rapamycin in an ALD model, the TG level was significantly reduced, and that after the autophagy was inhibited by the administration of chloroquine, TG levels rose again [144]. Interestingly, researchers subsequently observed similar phenomena in a disease model of NAFLD induced using a high-fat diet. These phenomena indicate that autophagy can alleviate liver injury by removing lipids in hepatocytes to some extent, in both alcoholic and nonalcoholic liver injury, likely through the same lipid autophagy mechanism.

Chronic drinking can inhibit proteasome activity and induce ER stress, resulting in protein aggregation and the formation of the Mallory-Denkosome (MDB) [145]. MDB is one of the characteristics of chronic ALD, which can be observed in patients with defects in autophagy or protein polymerization. Early studies showed that chronic alcohol consumption decreased the protein degradation rates of rat hepatocytes by 36%-40% [146] compared with the control group, which may be related to decreases in proteasome or autophagy activity. Studies have shown that liver lysosomes are alkalized following exposure to ethanol [147], and cathepsin contents decrease alongside this change [148]. In fact, in an MDB pathological mouse model, the use of rapamycin to induce autophagy was able to effectively eliminate MDB [149]. This indicates that autophagy may also be involved in the modulation of alcoholic MDB. It is noteworthy that, in addition to liver parenchymal cells, liver macrophages (Kupffer cells) responsible for autophagy attenuate alcoholic liver lesions by reducing ethanol-induced inflammation [150]. Overall, recent studies have generally proved that autophagy exerts an important protective effect in ALD through the clearance of compromised mitochondria, as well as accumulating lipid droplets and protein polymers.

## 4. The Digestive System

### 4.1. The Bowel

Abnormal intestine-specific immune regulation can lead to inflammatory bowel disease (IBD). IBD often takes a winding disease course, recurs frequently, and is difficult to cure. Studies have found that the mechanisms behind intestinal mucosal barrier subversion and intestinal inflammation are closely related to mitophagy [151]. IBD chiefly comprises ulcerative colitis (UC) and Crohn’s disease (CD).

#### 4.1.1. Ulcerative Colitis

Studies have shown that intestinal mucosal epithelial permeability increases in patients with UC, and that intestinal mucosal barrier damage may serve as an important indicator regarding the incidence of UC [152]. Maintaining the integrity of intestinal epithelial cells with regard to mucosal barrier function is closely tied to energy supply, and mitochondrial function may be essential to protect the mucosal barrier function of intestinal epithelial cells [153]. The pathogenesis of UC is regulated by both mitochondrial DNA (mtDNA) and nuclear DNA [154]. Mitochondrial dysfunction causes damage to intestinal epithelial cells (such as Paneth and/or goblet cells), which leads to decreased barrier function and increased permeability of the intestinal epithelium in turn stimulating intestinal inflammation.

The incidence and severity of UC are related to the increase in multiple autophagy proteins and ROSs. Several Atg proteins, such as Atg32 [155], Atg8 [156], and Atg11 [157], are intimately associated with the degradation of mitochondria. These act on the mitochondrial surface and promote the assembly of core Atg proteins within mitochondria, while the p62/ SQSTM1 complex combines with LC3 (the homologous protein of Atg8 on the surface of phagocytosed mitochondrial membranes) to induce autophagy bubbles and thus induce mitochondrial degradation. Studies have shown that dextran sulfate sodium salt (DSS) can induce the upregulation of tumor necrosis factor-α (TNF-α) and interleukin (IL)-1β expression in the colonic tissues of UC mice, alongside the downregulation of LC3B, p62 and Atg7 expression [158]. Both Atg7 and LC3B are often used to detect autophagy activity. The p62 protein is used as a substrate for autophagy and can be used as an indicator of autophagy.

Mitochondria are the main sites for ROS production and play central roles in cell death. High concentrations of oxidation molecules were detected throughout plasma, serum, and even exhaled gas and saliva samples of patients with UC, and the magnitude of UC was found to correlate positively with oxidative stress [159]. Mitochondrial biological function and homeostasis decrease as cellular oxidative stress rises, which fosters cellular damage, and ultimately kills cells [160]. Mitochondria are the main sites of intracellular ROS generation, but not the only one. At present, there is still some controversy surrounding the sources of ROSs that activate enteritis. However, some studies have shown that ROSs may also exert positive effects on the intestine [161].

#### 4.1.2. Crohn’'s disease

CD pathology can affect any part of the intestine, including the terminal ileum. Lesions can involve the deep intestinal wall. CD and UC have common disease susceptibilities and gene maps. Paneth cell damage and reduction are important mechanisms behind the intestinal pathology of CD. Khaloian et al. knocked out the 60 bp AU-rich fragment at the 3'end of the TNF gene in mice, to establish a mouse model of ileitis [162]. The results of the study showed that ileitis in mice was negatively correlated with a decrease of lysozymes in Paneth cells, as well as a decrease in the level of leucine repeat G protein-coupled receptor 5 in the crypt cells. A lack of HSP60 chaperones in intestinal stem cells can lead to mitochondrial metabolic disorders. Jackson et al. also found that mitochondrial dysfunction can lead to decreased antiproliferative proteins in Paneth cells, thereby increasing ileal inflammation in CD patients [163].

Inflammatory stimuli in the intestinal epithelial mononuclear phagocytes of CD patients originate from mutations to the reduced nicotinamide adenine dinucleotide phosphate-oxidase subunit [164]. Pierre et al. showed that the levels of mitochondrial proteins present in the ileums and colons of CD patients were remarkably lower than those of the general population [165]. The high mitochondrial activity of electrical signals in the ileums of CD patients is also related to defense against CD [166]. The OPTN protein is a 577 amino acid protein encoded by the *OPTN* gene, that is closely related to CD [167]. OPTN is a dependent receptor of the mitophagy PINK1-Parkin pathway. The promoter of the *OPTN* gene has a nuclear factor-κB binding site, which can initiate the expression of proinflammatory factors. Elimination of defective mitochondria can limit the participation of the inflammatory autophagy receptor p62 in nuclear factor-κB expression. However, studies have shown that p62 is not required in the PINK1-Parkin pathway [168].

The IL-10 receptor α gene, IL-10 receptor β gene, nucleotide-binding oligomerization domain protein 2 gene, X chromosome linked apoptosis inhibitor protein gene, apoptosis inhibitor protein 2 gene, immune-related GTPase family M protein 1 gene, and mutations to the X box binding protein 1 may all be involved in reduced mitophagy. Studies have shown that the X-linked inhibitor of the apoptosis protein gene mutation population is linked to predisposition to CD [169]. Nucleotide-binding oligomerization domain protein 2 gene mutations are also common in patients with CD [170]. The apoptotic inhibitor protein 2 and X-linked inhibitor of apoptosis protein can promote autophagosome-lysosomal fusion leading to mitochondrial autophagy [171]. A polymorphism of the autophagy gene Atg16L1 is related to CD. Pan’s cell loss occurs in intestinal mucosa that lacks Atg16L1, wherein TNF-mediated cell necrosis appears. The inflammatory response to IBD patterns can be reduced by TNF-α or receptor-interacting protein kinase suppressors [172]. Zhang et al. showed that a lack of Atg16L1 could lead to changes in macrophage function and aggravate CD [173]. Another research team knocked out the M protein 1 gene of the immune-related GTPase family in mice with DSS-induced colitis and found that this gene may modify drastic inflammatory responses in the intestinal tracts of mice by regulating mitophagy [174].

## 5. The Urinary System

### 5.1. Kidney Fibrosis

Autophagy, an important cellular defense mechanism, is a tightly conserved evolutionary process that has a close connection to nephrofibrosis. It was recently shown that valproic acid may suppress nephrofibrosis in mice with unilateral ureteral obstruction (UUO), by inducing autophagy [175]. Fibroblast growth factor 2 (FGF2) and fibronectin produced after UUO enhance renal fibrosis [176]. Xiao et al. found in cultured renal fibrosis cells as well as in animal models that autophagy is activated to promote defense against ROSs and reduce related protein fibronectin and type I collagen, thereby alleviating renal fibrosis both *in vitro* and in animal models [177]. It has also been observed that 3 MA inhibition of autophagy boosted renal tubular cell apoptosis and interstitial fibrosis in an animal model of renal fibrosis [178].

Autophagy can not only inhibit renal fibrosis but also promote renal fibrosis. Livingstone et al. found that in a UUO-induced mouse renal fibrosis model, the autophagy of renal tubular cells was continuously activated to promote renal fibrosis [133].

It has been shown that autophagy is activated in blocked renal tubules, which manifests as autophagy volume aggregation, increasing Becline-1 expression, and the transformation of LC3-I to LC3-II. These changes were found to be concomitant with an increase in autophagic lysosomes and the enhancement of their activity, further indicating that autophagy is enhanced in blocked renal tubules. Autophagy also induces the apoptosis of renal tubular cells in blocked renal tubules. Importantly, the development of renal tubular atrophy is time-dependent with autophagy and apoptosis, suggesting that autophagy may work in conjunction with apoptosis to induce renal tubular atrophy and renal unit loss [133].

### 5.2 Acute Kidney Injury

The pathogenesis of acute kidney injury (AKI) involves several aspects, including cellular famine, shortage of oxygen, ER stress, and oxidative damage. These reactions can activate the autophagy signaling pathway.

In the pathogenesis of AKI, cellular stress can effectively trigger autophagy. The generation of ROSs and the ensuing oxidative stress are involved in the onset and advancement of AKI. At the same time, ROSs can also trigger autophagy [179, 180]. Renal mitochondria are susceptible to ROSs, which can induce active oxygen species, resulting in mitochondrial malfunction and loss of membrane potential triggering autophagy. In clinical research and animal models, ER stress was found to be involved in autophagy. ER stress induced by tunicamycin activates an unfolded protein response against renal ischemia-reperfusion (IR) [181]. IR damage has been found to induce autophagy in animal models [182-184]. In a rat model of renal IR injury, the expression of the autophagy-related proteins Beclin-1 and LC3 in renal tubular epithelial cells increased significantly [181]. Kimura et al. found that the presence of LC3 in the proximal kidney tubular cells of Atg5 deficient mice is reduced after renal IR injury, and wherein autophagy substrate accumulation and increased serum creatinine and urea nitrogen levels were also observed. This suggests that basal autophagy has a protective effect on renal injury induced by renal IR injury [179]. Similar results were found in the proximal tubular Atg7 knockout rats. Compared with wild-type rats, the proximal tubules and renal function of rats with who had their Atg7 genes knocked out are more severely affected by IR injury [185]. The progression regime of autophagy adjustment within in case of renal IR injury remains unclear. MTORC1 negatively modulates autophagy mediated by the phosphorylation of ULK1 and is also engaged in a broad spectrum of cellular processes such as promoting cell growth, augmentation, survival, and metabolism [186-188]. Knockout of mTORC1 in the proximal tubule can increase the susceptibility of the kidney to IR injury, which may be due to the inhibition of mTORC1, which enhances autophagy activity. This also reduces the growth and survival rates of cells [188, 189].

Autophagy has a protective effect on kidneys in cases of cisplatin-induced AKI. In proximal tubule epithelial cells, the autophagy inhibitor 3-MA or siRNAs targeting Beclin-1 and Atg5 increased the cisplatin-induced activation of apoptotic enzymes and apoptosis, suggesting that autophagy protects the kidney from cisplatin-induced AKI [190]. Chloroquine can effectively block autophagy flux, which can, in turn, aggravate cisplatin-induced AKI and harm renal function [182, 183]. This further illustrates the protective properties of autophagy against cisplatin-induced renal injury.

## 6. The Circulatory System

### 6.1. Aplastic Anemia

Aplastic anemia (AA) is a malignant hematological disease of bone marrow hematopoietic failure, characterized by a reduction in the proliferation of bone marrow hematopoietic stem cells and a reduction of whole blood cells in the peripheral blood. Its main clinical manifestations are anemia, bleeding, and infection. It has a relatively high mortality rate. AA is related to hematopoietic stem cell injury, hematopoietic microenvironment injury, and immune system changes. In recent years, the effect of autophagy is gradually attracting more attention alongside research into the role of autophagy in malignant hematologic diseases and its contribution to the pathogenesis of AA.

Huang et al. studied levels of autophagy in patients with AA versus healthy controls. LC3-II, an autophagy-specific marker, was found to be present in markedly lower levels in hematopoietic stem/progenitor cells from patients with AA compared with healthy controls, while patients with severe AA had lower levels than those with less-severe AA [191]. This suggests that hematopoietic stem/progenitor cells in patients with AA are deficient in terms of their autophagy-related functions, and that autophagy, as a cytoprotective mechanism, may be an important reason behind the reduced abundance and polarization of hematopoietic stem/progenitor cells in patients with AA. Huang et al. investigated the levels of autophagy present in the bone marrow stem/progenitor cells of three types of patients with AA following immunosuppressive therapy. The patients either had no remission, partial remission, or complete remission. They found that although levels of the autophagy-specific marker LC3-II were still relatively low compared with those of the healthy control group, they gradually increased as the level of remission increased, suggesting that autophagy defects in the bone marrow stem/ progenitor cells of patients with AA can gradually recover as the disease is managed. This suggests that autophagic defects in hematopoietic stem/progenitor cells may partially explain the pathogenesis of AA.

The microenvironment of hematopoietic stem cells Is an anoxic one, and its metabolism remains relatively static, mainly relying on glycolysis for energy supply. Approximately every five months, self-renewal is performed to ensure the hematopoietic reserve, to avoid the excessive accumulation of oxidative products, damaged organelles/proteins, and other metabolic wastes, as well as to avoid the self-exhaustion caused by excessive proliferation. From static to directional differentiation into various lineages of blood cells, oxidative phosphorylation gradually produces ROSs, damaged organelles, and other metabolic wastes. By knocking out the Atg7 autophagy gene in the hematopoietic stem cells of mice, Mortensen et al. found that massive amounts of mitochondria and ROSs accumulated in their cells, and that the cells exhibited highly abnormal behaviors. They began to proliferate and differentiate continuously, as well as lose normal hematopoietic function [192]. Another study by Lee et al. showed that the autophagy-related protein Atg7 was able to regulate the transcription of the cell cycle inhibitor P21 by binding to the tumor suppressor protein P53 [193]. Moreover, it was observed that cells lacking the Atg7 autophagy gene could not enter the static state of cycle arrest when they were hungry to avoid the adverse environment, indicating that autophagy is of crucial importance for keeping blood-forming stem cells in a static state.

Liu et al. showed that after knocking out the gene that encodes the FIP200 protein in the ULK1 complex of mice hematopoietic stem cells, resulting in multiple autophagy-related defects, hematopoietic stem cells accumulated substantial amounts of mitochondria and ROSs [194]. The cells begin to proliferate excessively, leading to cell depletion. There was also an imbalance in the differentiation of myeloid cells: there was a large increase in the number of granulocytes, and the proportion of lymphocytes decreased significantly. These results suggest the involvement of autophagy in the multidirectional proliferation and derivatization of hematopoietic stem cells as well.

The hematopoietic microenvironment is the place where hematopoietic stem cells proliferate, differentiate, and mature, and is intimately involved in hematopoietic function. Disturbances in the hematopoietic microenvironment can induce the abnormal proliferation and differentiation of blood-forming stem cells, resulting in the occurrence of AA. In the hematopoietic microenvironment, bone marrow mesenchymal stem cells can differentiate into adipocytes, endothelial cells, and osteoclasts. Adipose cells can secrete negative hematopoietic regulatory factors such as neuropillin 1 and TNFα. The balance in the numbers of these signalers is crucial to maintaining the health of the hematopoietic microenvironment. Autophagy regulates the rate of adipogenesis in bone marrow mesenchymal stem cells. Wang et al. reported that rapamycin, given as an autophagy inducer, induced bone marrow mesenchymal stem cells in patients with AA [195]. Suppressing the activity of the mTOR protein reduced adipogenic differentiation, controlled the number of adipocytes in the medulla, reduced the negative hematopoietic regulation caused by adipocytes, improved the bone marrow microenvironment, and promoted appropriate colonization and differentiation in hematopoietic stem cells. It has also been suggested that autophagy deficiencies in the medullary cavity are likely to affect the balance in adipogenicity in bone marrow mesenchymal stem cells, causing increases in adipocytes, negative regulation of hematopoiesis, and abnormal hematopoietic microenvironments. This, in turn, promotes the occurrence of AA and other related blood diseases.

Over the past few years, several studies have suggested that autophagy also affects the steady-state regulation of cellular immunity. Abnormal autophagy can disrupt the stability of the immune system, thereby promoting the occurrence and development of immune-related diseases. Cytotoxic T cells (Tc) are abnormally regulated in patients with AA, leading to their numbers increasing significantly. Abnormally high levels of Tc cells can promote the secretion of TNFα, INFγ, IL2, and other negative hematopoietic regulatory factors, thereby inhibiting the hematopoietic function of the bone marrow [196, 197]. In addition, the number of Th1 subtype of Helper T cells (Th) in patients with AA can increase abnormally, while the number of Th2 subtypes of other types do not change significantly, leading to an imbalance in the Th1/Th2 ratio. The dynamic balance of Th1/Th2 is essential to preserving normal immune responses in the body. Abnormally high levels of Th1 cells not only promote the expression of negative hematopoietic regulatory factors such as TNF-α and INF-γ downstream, but also interact with suppressor T cells (Ts). This indirectly inhibits the differentiation of B lymphocytes and the killing function of Ts cells, thereby inhibiting both humoral and cellular immunity, directly or indirectly limiting the abilities of stem cells to function as hematopoietic cells, eventually leading to hematopoietic failure in the bone marrow.

## 7. The Reproductive System

### 7.1. Polycystic Ovary Syndrome

Polycystic ovary syndrome (PCOS) is a disorder of endocrinology and metabolism that occurs among women. The prevalence of PCOS is increasing annually. Its main features are increased androgen, ovulation disorders, and polycystic ovary changes, often accompanied by insulin resistance (IR) and compensatory hyperinsulinemia, obesity, dyslipidemia, and low-grade chronic inflammation. Long-term development can lead to increased incidence of diabetes, immune system disorders, cardiovascular diseases, and even gynecological tumors. The etiology of PCOS is complex, involves multiple organs, and is affected by multiple factors such as genetics, environment, and lifestyle. At present, the pathogenesis of PCOS is still not entirely clear, which poses a great challenge to its clinical treatment [198-200]. In recent years, there has been a growing recognition of the role that autophagy plays in metabolic disorders pertaining to PCOS. The ovaries are the main organs that develop lesions during PCOS and are also the main sources of high androgen in patients with PCOS. Recently, it has been shown that ovarian granulosa cell autophagy increased significantly in rat models of PCOS [201].

Studies have confirmed that both patients with PCOS and rat models of the disease have higher levels of autophagy in their ovarian tissues. The core regulators of autophagy that upregulate ovarian granulosa cells in PCOS are Beclin 1, LC3, and LC3-II/LC3-I. As these increase, the levels of autophagy substrate SQSTM 1/p62 decrease [202]. Correlation analyses have shown that the level of Beclin-1 is positively correlated with serum testosterone (T) and anti-mullerian hormone (AMH). In addition, ovarian granulosa cells express Toll-like receptor 4 (TLR4) and TLR9, which are regulated by luteinizing hormone (LH) and T, respectively, through the TLR/myeloid differentiation factor (MyD88) signaling pathway, which induces autophagy [78]. These findings suggest that the abnormal activation of autophagy is presumably a factor in the emergence and progression of PCOS and in the production of androgens in PCOS, which may in turn be a cause of follicular development disorder. In patients with PCOS, the endometrium is another primary location of lesions. Research has shown that there are substantial differences in endometrial autophagy levels at different stages of the menstrual cycle in healthy women of childbearing age. LC3-I and LC3-II are much higher in the secretory phase (high levels of both estrogen and progesterone) than in the proliferative phase (high levels of estrogen only) and are regulated by ovarian hormones, participating in the apoptosis of endometrial epithelial cells and regulating the cyclical changes of the endometrium [203]. At present, hormonal disorders cannot fully explain the abnormal endometrial changes seen in patients with PCOS. It has been shown that *FOXO1* and the autophagy-related genes *ATG13*, *ATG14*, *Beclin-1*, *ATG3*, *ATG5*, and *GABARAP* are significantly decreased in patients with PCOS. Free T levels are inversely correlated with *ATG13* and *ATG14*, and *FOXO1* is positively correlated with Beclin-1. In addition, both *FOXO1* and *Beclin-1* levels are significantly altered in different pathological types of PCOS endometria. Lower levels of *FOXO1* and *Beclin-1* correlate with worse pathological types of endometria, worse prognoses, and a higher likelihood of developing endometrial cancer [204]. Studies have shown that the autophagy markers Beclin-1 and LC3 are significantly increased in patients with non-PCOS endometrial cancer, and that elevations in their levels correlate positively with tumor grade and myometrial invasion, and negatively with 5-year survival rate [205]. This is not consistent with findings pertaining to PCOS endometrial lesions, indicating that there are differences in the mechanisms behind endometrial cancer in PCOS patients *vs* non-PCOS patients and that autophagy might play an essential role. In addition, disorders in autophagy regulation may lead to the disappearance of the change cycle of endometrial regularity and imbalances in endometrial secretion-proliferation, which may be related to endometrial receptivity.

IR is the most important metabolic change of PCOS, which affects 50%-70% of PCOS patients. It mainly involves two signaling pathways: PI3K/Akt/mTOR and MAPK/ERK. It can cause compensatory hyper-insulinemia, promote the production of PCOS and ovulation disorders, participate in the occurrence and progress of PCOS, and affect the function of multiple organs [206, 207]. A pivotal aspect of autophagy is in the regulation of pancreatic function and the regular function of insulin target structures including skeletal muscle, liver, and fatty tissue. Insulin has been shown to enable the activation of mTORC1 to quench ATG13 and activate PKB, to inhibit the ATG gene. Insulin inhibits autophagy by regulating these two pathways, thereby inducing IR [208]. In a mouse model of IR induced by a high-fat diet, autophagy in liver cells was impaired, mTORC1 levels in the skeletal muscle were significantly increased, and autophagy signal transduction was inhibited. After promoting the presentation of ATG7, the levels of Beclin-1, ATG5, ATG12, and LC3 increased, and the IR disease status improved significantly [209, 210]. Therefore, it can be said that the correlation between IR and autophagy is potentially related to PCOS metabolic disorder.

About 35%-60% of PCOS patients are obese, and adipose tissue has complex endocrine and metabolic functions, that are related to many diseases. In PCOS, obesity aggravates the degree of IR, the high androgen state, and reproductive dysfunction. It is also closely related to long-term complications of PCOS such as diabetes and cardiovascular diseases. The maintenance of the metabolic function of adipose tissue requires the coordination of multiple pathways, among which autophagy regulation is considered an important one. The occurrence of IR in obese PCOS patients is related to excess leptin and tumor necrosis factor-α (TNF-α) produced by adipose tissue. These two factors can inhibit insulin receptors by regulating the phosphorylation of IRS-1, or interfere with insulin by inhibiting signal transmission through PRAR-γ [211]. Changes that have been observed in the adipocytes of obese individuals include high expression levels of the autophagy markers LC3-II and ATG5, significant decreases in the levels of mTOR expression, and higher LC3-II levels that correlate positively with BMI index, suggesting that adipocyte autophagy in is active in these individuals. Other studies have found that after the autophagy inhibitor chloroquine interferes with 3T3-L1 adipocytes, the levels of PRAR-γ, the main transcription factor regulating adipocyte differentiation, drop as this factor inhibits adipogenic differentiation. Yoshizaki et al. found that autophagy-related genes such as Atg5 were reduced *in vitro* in cultured 3T3-L1 adipocytes, suggesting that autophagy is inhibited under obesity conditions [212]. This indicates that autophagy has a complex function with regard to regulating the activity of adipose tissue.

## 8. Cancer

Autophagy plays a major role in maintaining cellular homeostasis. It is also intimately associated with cell proliferation, differentiation, and senescence. Recently, it has been recently found that dysfunctional autophagy has already been described among many different types of diverse malignant tumor cells (including lung carcinoma, hepatocellular carcinoma, breast carcinoma, and so on). During the process of tumor development, autophagy exerts a dual effect on both tumor promotion and inhibition. Autophagy can inhibit tumor growth at the initial stage of tumor occurrence; however, it can also provide energy to starved tumor cells. The clarification of the relationships between autophagy and tumor cells is therefore of great importance, autophagy, and tumor cells, especially with regard to the oncogenesis and progression of tumor cells.

### 8.1. Autophagy Promotes Tumorigenesis

The promoting effect of autophagy on tumors mainly manifests in multi-drug resistance and the reduction of sensitivity to radiation. The causes are complicated, mainly including biochemical and physiological factors, such as insufficient local blood perfusion and changes in the tumor microenvironment. Multidrug resistance often develops in tumors as a result of chronic chemotherapy, contributing to refractory tumors and tumor relapse. Chemotherapeutic drugs were discovered to inhibit the PI3K-Akt-mTOR signaling pathway, upregulate AMPK activity, and induce autophagy in tumor cells, while miR-27b-3p inhibits autophagy and re-sensitizes drug-resistant colorectal cancer cells to oxaliplatin [213, 214]. Radiation therapy can destroy the DNA structures of tumor cells and influence their proliferation.

The PI3K-AKT-MTOR pathway is considered to be an approach (or breakthrough point) for exploring autophagy and resistance to radiotherapy and chemotherapy in the field of oncology.

In tumor cells, autophagy can promote tumorigenesis by degrading tumor inhibitors. For example, the upregulation of CMA accelerated the degradation of tumor suppressor MST1 in patients with breast cancer [215]. Tumor cells can also inhibit apoptosis through autophagy [216]. CMA can decrease the activity of PKM2 and mutant p53 to strengthen Warburg effects to provide energy for tumor cells [217]. The metabolite produced by autophagy can also be used to promote tumor development and metastasis [218].

### 8.2 Autophagy Inhibits Tumorigenesis

The identification of drugs that elicit autophagic cell death to combat neoplasia cell proliferation has become a topic of much interest in recent years. Several studies have proven that autophagic death can be regulated by intracellular components [219, 220]. It has been found that several antineoplastic agents can inhibit tumor cells by targeting the autophagic pathway [221]. Some tumor-related proteins can also be degraded by autophagy, inhibiting tumor metastasis [222, 223].

## 9. COVID-19

COVID-19 is caused by the SARS-CoV-2 virus infection that has caused incalculable damage to the economy, health, and lives of people worldwide since the outbreak in late 2019. Worldometers World Live statistics show that the cumulative number of patients diagnosed with COVID-19 and the number of deaths worldwide have surpassed 500 million and 6 million, respectively, as of April 14, 2022. SARS-CoV-2, a highly contagious coronavirus, is transmitted by droplets and aerosols, causing lung disease and even life-threatening diseases [224]. Similar to other types of viruses, coronaviruses may use components of the cellular autophagic process to inhibit degradation and promote replication [225]. However, experimental proven that autophagy can affect SARS-CoV-2 virus replication is very limited. Numerous studies have shown a close connection between coronaviruses and autophagy. Coronaviruses probably involve in the entire autophagenesis procedure in human cells, while autophagy may also partially take in the entire infectious process of coronaviruses [226, 227]. The SARS-CoV-2 virus is involved in cellular autophagy mainly by affecting the synthesis of omegasome intermediates, inducing phagocytosis, and inhibiting vesicle nucleation [228]. By using proteomic techniques, Bouhaddou et al. found that the autophagy-related p38/MAPK signaling pathway is activated in SARS-CoV-2-infected cells, while the autophagy-related PIK3CA/AKT signaling pathway was inhibited [229]. Various metabolic pathways involving glycolysis and protein translation are interfered with by The SARS-CoV-2 virus through inhibition of the activated AMPK signaling pathway, ultimately inhibiting the initiation of cellular autophagy [230]. SARS-CoV-2 virus inhibits the occurrence of autophagy in human-infected cells, which translates into an increase in the rate of apoptosis [231]. Several studies exploring the effects of autophagy-related drugs on the SARS-CoV-2 virus found that cellular autophagy can promote SARS-CoV-2 infection and replication to some extent. Inhibitors of the p38/MAPK signaling pathway show good antiviral ability in cells infected with SARS-CoV-2 [229]. Gassen et al. found that autophagy-related agonists including spermidine, are able to inhibit the spread of SARS-CoV-2 in infected cells by inducing the appearance of cellular autophagy [230]. Existing studies only demonstrate that cellular autophagy is associated with SARS-CoV-2 virus infection, but its specific molecular mechanisms and effects are not yet clear. As autophagy inhibitors, chloroquine and hydro-xychloroquine showed excellent anti-SARS-CoV-2 ability in cellular experiments [232, 233]. Although their anti-SARS-CoV-2 were not validated in subsequent animal and human experiments, they still have potential possibilities for treating COVID-19 by autophagy[234-236].

## 10. Autophagy with mitochondrial dysfunction in diseases

In some diseases, there may be certain oxidative reactions or oxidative factors produced in the body to which mitochondria are susceptible, which can lead to mitochondrial dysfunction and trigger autophagy, allowing further disease progression. In cells with autophagy, normal mitochondrial recycling is the basis for maintaining healthy organelles. In autophagy-deficient cells, the accumulation of dysfunctional mitochondria leads to elevated levels of reactive oxygen species (ROS), which sustainably activate SIRT and PARP family stress-responsive enzymes. The uncontrolled cleavage of Nicotinamide adenine dinucleotide (NAD) by these enzymes leads to the depletion of the intracellular NAD pool, and the loss of available NAD negatively affects mitochondrial metabolism, triggering mitochondrial inner membrane (IMM) depolarization and apoptosis [237, 238].

### Conclusion

Autophagy is an important regulation mode of eukaryotic cell homeostasis, which is instrumental in ensuring the metabolism of the organism in its regular physiological condition. While the organism is in a pathological state, for instance, when starving, aging, and external stimulation, autophagy is activated by a series of complex molecular signals. The appropriate enhancement of autophagy is an active non-invasive response of cells to stress. At this time, if autophagy is relatively insufficient, the damaged organelles and waste proteins may not be degraded and utilized in time, resulting in damage.

Autophagy has diverse functions in different diseases, and different types of autophagy also have different roles in diseases. Therefore, clarifying the mechanism of autophagy in the onset and evolution of disease helps propose new therapeutic strategies and provide new ideas for disease treatment. Although there are numerous ongoing investigations about the function of autophagy in illness, further studies are still needed to comprehensively analyze the detailed functions of autophagy in the induction and progress of related diseases, so as to provide direction and evidence for clinical prevention and treatment and improve the cure rate of diseases.
